# agINFRA: a research data hub for agriculture, food and the environment

**DOI:** 10.12688/f1000research.6349.2

**Published:** 2015-06-15

**Authors:** Andreas Drakos, Vassilis Protonotarios, Nikos Manouselis

**Affiliations:** 1 Polytechnic Building, University of Alcala, Barcelona, 28871, Spain; 2 Agro-Know, 152 35 Vrilissia, Greece

**Keywords:** agri-food, cloud-based, grid-based

## Abstract

The agINFRA project (www.aginfra.eu) was a European Commission funded project under the 7th Framework Programme that aimed to introduce agricultural scientific communities to the vision of open and participatory data-intensive science. agINFRA has now evolved into the European hub for data-powered research on agriculture, food and the environment, serving the research community through multiple roles.

Working on enhancing the interoperability between heterogeneous data sources, the agINFRA project has left a set of grid- and cloud- based services that can be reused by future initiatives and adopted by existing ones, in order to facilitate the dissemination of agricultural research, educational and other types of data. On top of that, agINFRA provided a set of domain-specific recommendations for the publication of agri-food research outcomes. This paper discusses the concept of the agINFRA project and presents its major outcomes, as adopted by existing initiatives activated in the context of agricultural research and education.

## Introduction

agINFRA was an innovative Integrated Infrastructure Initiative (I3) project (2012–2015) that aimed to introduce agricultural scientific communities to the vision of open and participatory data-intensive science. To achieve this aim, agINFRA designed and developed a scientific data infrastructure for agricultural sciences that facilitates the development of policies and services that promote the sharing of data among agricultural scientists in a manner that develops trust within and amongst their communities. The key goals and corresponding project objectives are as follows:

- Increased sharing and federation of agricultural data (scientific objective)
  – Successfully deploy an open infrastructure for the sharing of digital agricultural content;
  – Include in the infrastructure resources ranging from raw observational and experimental data through to publications;
  – Promote the sharing of data amongst the wider scientific community to build trust;
  – Ensure outcomes significantly advance the state-of-the art in agricultural e-infrastructures;
- Efficient data management in the agricultural research process (scientific objective)
  – Ensure stakeholder needs are met with regards to data management and sharing;
  – Involve as many agricultural data sources as possible to provide maximum value;
  – Facilitate easier curation, certification, annotation, navigation and management of data;
  – Create new opportunities for data intensive research in the agricultural domain
- Deployment of robust European service infrastructure for scientific agricultural data (technical objective)
  – Deploy tools (exchange standards, software, methodologies) for collaboration between European institutions in data-intensive research;
  – Validate the approach by enabling users to interact with each other and the data
  – Identify gaps in standards that will be needed to guarantee the integrity/authenticity of data
  – Establish specific and realistic indicators to measure success
- High interoperability between agricultural and other data resources (technical objective)
  – Improve interoperability between existing e-infrastructures;
  – Successfully interconnect agricultural data repositories through extended metadata;
  – Advanced implementation and adoption of European standards and specifications

agINFRA has now evolved into the European hub for data-powered research on agriculture, food and the environment, and it is currently serving the research community through multiple roles, including (but not limited to) the following:

- A research data sharing help desk and service point for Horizon 2020 projects in the aforementioned thematic areas;
- The agri-food node of OpenAIRE ( https://www.openaire.eu);
- A big (research) data machine and stack of technologies;
- An ecosystem for research data scientists, developers and startups.

## agINFRA outcomes

The agINFRA project has been seen as a project that would bring a number of advances in the area of agricultural data management and re-usage. Its main goals as they had been set up from the beginning of the project and revised during the project lifetime can be summarized as ^1^:

- Set up FAO and its stakeholders as the managing and promoting stakeholder of a data infrastructure for agricultural scientists, enhancing their current reach with projects as the CIARD Routemap to Information Nodes and Gateways (RING - http://ring.ciard.net), AGRIS ( http://agris.fao.org/) and the Agriculture Information Management Standards (AIMS - http://aims.fao.org/);
- Connect existing networks of data repositories and institutional/national repositories through this data infrastructure, enabling new possibilities for retrieval and data analysis;
- Connect this data infrastructure to other infrastructures (such as LifeWatch - http://www.lifewatch.eu/ and ViBRANT - http://vbrant.eu/) and data repository networks (such as VOA3R - http://voa3r.eu/ and Organic.Edunet - http://www.organic-edunet.eu, Integrated capture information system by D4Science - ICIS http://www.d4science.eu/icis) to aggregate and expose their data resources through a flexible infrastructure;
- Provide mediating capabilities for heterogeneous, distributed data sets in agriculture, including the necessary scalability and high performance support to handle complex queries and data extraction processes;
- Provide machine-processable interfaces to the data resources and systems integrated, enabling the development of semantics-aware applications and using the recommendations widely adopted for open linked data;
- Further integrate the educational content repository stakeholders, allowing agricultural scientists to have access to and share their data resources that they used for education/extension. To achieve its aims, the agINFRA project took upon a number of initiatives to help shape a new area around agricultural data management.

### agINFRA stakeholders analysis

One of the key elements of the agINFRA analysis that took place during the first years of the project was the stakeholders analysis. We have in several project reports stated that the main prospective “users” of the agINFRA infrastructure are data managers, information service managers and information system developers dealing with agriculture-related data and more precisely:

- Research projects, i.e. researchers and their IT/information managers: This group is of interest mainly as end-users who will use portals supported by agINFRA to access content and data/datasets. Researchers are also providers of content/data which, however, should be deposited in their institutional repositories or national/international subject or domain based repositories from which the metadata is collected into the agINFRA data layer;
- IT/information managers of individual research organisations (e.g. university departments, research centres, research libraries, etc.) who manage content/data repositories and information services, from which metadata is collected in the agINFRA data layer;
- IT/information managers of shared discipline- or subject-based content/data repositories and services, which are content/data aggregators and service providers on top of the local institutional repositories. This category also includes national or international agencies that aggregate research data and make it available for purposes such as agricultural geographic and other information systems (e.g. for land use planning, conservation of natural resources, risk assessment, etc.);
- Developers/providers of software-based applications for research and content/data management tasks who are interested to customize or further develop agINFRA components, tools and services. They may become involved in agINFRA-related, open source software development or simply use agINFRA Application Programming Interfaces (APIs) to create useful applications such as mash-ups of research data;

This stakeholder analysis was essential to understand the potential users of agINFRA, understand their needs and proceed with the deployment of an infrastructure that will serve the actual needs of the agricultural community. The detailed analysis that was done, helped the project partners shape the agINFRA common vision ^1, 2^.

### agINFRA common infrastructure

**Figure 1.**
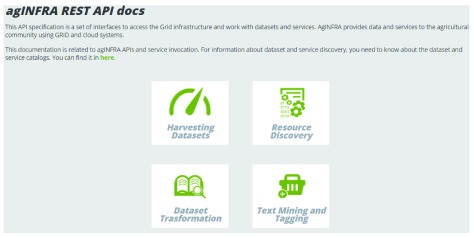
Documentation of the agINFRA REST API. Source: agINFRA website ( www.aginfra.eu).

From the beginning of the agINFRA project, it was promised to develop and deliver a shared technology infrastructure. During the lifetime of the project, agINFRA partners designed and deployed research infrastructure components and APIs (Application Profile Interfaces) that support the agINFRA applications and layers in a federated way. The agINFRA Virtual Organisation (VO) has been created to organise the infrastructure provided by the agINFRA partners (Institute of Physics Belgrade - IPB, Instituo Nazionale di Fisica Nucleare - INFN, Computer and Automation Research Institute of the Hungarian Academy of Science - SZTAKI) but also to connect with external infrastructure providers as for example the Greek Research and Technology Network (GRNET), the National Centre for Scientific Research ‘Demokritos’ (NCSR) or other National Grid Initiatives through the European Grid Initiative network. The “vo.aginfra.eu” Virtual Organization (VO) supporting the agINFRA community is operated on the European Grid Infrastructure (EGI) infrastructure, it is supported by all Grid sites participating in the project (AEGIS01-IPB-SCL, INFN-CATANIA, INFN-ROMA3 and SZTAKI), and it is delivering access to more than 2000 CPU cores and 900 TB of storage space to its users. The primary agINFRA Virtual Organization Membership Service (VOMS) server (voms.ipb.ac.rs) is installed at the IPB. The name vo.aginfra.eu is chosen with a format recommended by EGI, and allows global VO registration at CIC portal that will certainly increase VO visibility ^3^. On top of the agINFRA infrastructure a number of application have been ported to the Grid infrastructure (Grid modules) with a RESTful interface on top that enables configuration of application input parameters and output retrieval. For each of the Grid modules, the applications had to be gridified and exposed through a RESTful API ( Figure 1). All the ported applications are related with the aggregation and management of data (e.g. agHarvester) and the list of the services is available through the API section of the agINFRA site ( www.aginfra.eu/api).

In addition agINFRA took advantage of two different Science Gateways (SG) architectures for the management of complex job workflows and on user membership management through the adoption of the Identity Federations and Identity Providers approach for access to Grid and/or Cloud resources. Both of the generic versions of the SG have been customized to support the needs of the agricultural research data community. Finally, one of the biggest risks in e-infrastructure projects is the sustainability and compatibility of the infrastructure elements. From the beginning of the project, the agINFRA infrastructure was created in a way to be fully compatible with the EGI Federated Cloud. Being compliant with such an initiative allows the easy of new resources to be committed to the project. In addition, EGI itself has recognized the work and the needs of the community and during the upcoming EGI-ENGAGE project (the flagship project of EGI) a Virtual Team for the Agricultural Sciences is to be created. At the same time, additional initiatives have shown the will to provide resources and support the work of the agINFRA as for example the pledge for a Linked Open Data layer for the agricultural community.

**Figure 2.**
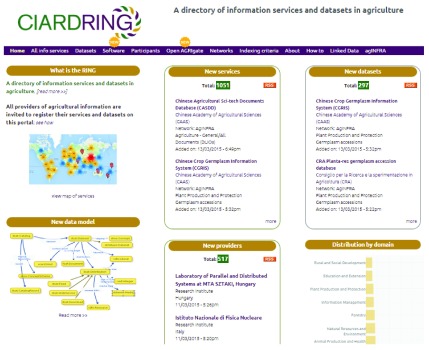
The CIARD RING home page. Source: CIARD RING ( http://ring.ciard.net).

### CIARD-RING registry of information services and datasets

One of the main advances during the agINFRA project was the revamp of the CIARD RING ( http://ring.ciard.net) global directory of web-based information services and datasets for agriculture. The CIARD RING, a project maintained within the CIARD initiative and is led by the Global Forum on Agricultural Research (GFAR), has become a machine-readable hub/switchboard to information sources such as search engines, databases, repositories, Open Archives, feeds, data sheets etc ( Figure 2). All information stored/registered in the RING i.e. metadata about services and datasets, can be queried via SPARQL and via a simpler REST API. In addition, during the agINFRA project, it has become evident that in order for the RING to become the ‘central broker of the information’ around the agricultural community, even more additions had to be made. Today, the RING includes a registry of organizations and networks with the vision of being part of the future AgriProfiles list of people, networks and organizations in the agricultural research. Starting from the agINFRA project, software are also registered in the RING as a way to easily find and retrieve software services (tools, web services) that can process datasets. Parallel with the RING evolution, the AIMS Vocabularies, Metadata Sets and Tool (VEST) registry ( http://aims.fao.org/vest-registry) was also enhanced as a catalog to retrieve datasets, metadata sets, tools and Knowledge Organization Systems (KOS) used in the broader agricultural information management community. Last but not least, during the lifetime of the project, project partners registered their own datasets or collections in the RING enhancing the number of available datasets especially for the case of bibliographic, educational, germplasm and soil datasets.

### Data Interoperability and Linked Open Data

The agINFRA project specifically aimed to create an environment to support data interoperability. One of the main outcomes of the data interoperability work was the generic aggregation workflow and the common metadata model. As it has been show cased in the project, a very well designed, domain specific aggregation workflow can be used to aggregate data from different sources. This generic version of the aggregation workflow was used and tested under the prism of the agINFRA services (i.e. AGRIS, Green Learning Network - GLN). As it was specified, part of the workflow was the transformation of the aggregated metadata records to an internal format. The usage of such format allows easily managing and re-using the data. But the work around the data interoperability didn’t stop in the aggregation of metadata. During the agINFRA project a lot of effort was made on the implementation of a Linked Open Data layer, especially for the case of germplasm and soilmaps data ( http://vocabularies.aginfra.eu). The work done in the agINFRA project set the first steps for the Global agricultural Concept Scheme (GACS) project that aims to create a hub for thesauri in the agricultural field, in multiple languages, for use in Linked Data, a project supporter by The Food and Agricultural Organization of the United Nations (FAO) responsible for the AGROVOC Concept Scheme, CAB International (CABI) responsible for the CAB Thesaurus (CABT), and the National Agricultural Library of the USA (NAL) managing the NAL Thesaurus.

### agINFRA European Open Data Node

One of the obligations of the agINFRA project was to participate in the FP7 pilot program that requires all project publications to be openly available and delivered in OpenAIRE ^2^. OpenAIRE ( https://www.openaire.eu), a three-year project, establish the infrastructure for researchers to support them in complying with the European Commission Open Access pilot and the European Resuscitation Council Guidelines on Open Access. It delivers an electronic infrastructure and supporting mechanisms for the identification, deposition, access, and monitoring of FP7 and ERC funded articles. With the pilot program during FP7 becoming mandatory for all projects funded under the Horizon 2020 framework, agINFRA saw the need and opportunity to provide its data management services for projects and researchers in the agri-food community. Hence, in collaboration with OpenAIRE, the thematic agINFRA European (EU) Node was created as an aggregator that will collect all information for the agri-food sector and supply it to OpenAIRE relieving its contributors from the duty from registering themselves. To support the agINFRA EU Node, the agINFRA project in collaboration and with the support of AIMS and CIARD introduced a set of recommendations applying to agri-food research community for data management, sharing and dissemination aiming among others to provide a framework for the research community of European agri-food research institutions that need to follow the H2020 Open Access mandate and share their metadata. These recommendations are: 1) Metadata repository for data sources and publications following international metadata standards and classification schemes:

- Specific metadata standard (e.g. AGRIS metadata application profile, Dublin Core) describing publications (e.g. author, abstract, keywords);
- Specific metadata standard (e.g. CERIF: Common European Research Information Format) describing datasets (e.g. processed data of experiments);
- Specific classification schemes according to the needs of each research community (e.g. AGROVOC terms ( http://aims.fao.org/agrovoc)

2) Repository registration in an agricultural aggregator (like the CIARD RING: http://ring.ciard.net) 3) Institutions’ researchers’ profiles and/or Institutions’ profiles need to be publicly available and accessibly by the agri-food community (using tools like registration in the AgriVIVO: http://www.agrivivo.net or the future AgriProfiles) 4) Data and publications accompanied by licenses using an international common standard (like Creative Commons: http://creativecommons.org) 5) Sharing the (metadata) descriptions of institutions data/publications with the agri-food research community (like the European AGRIS node “agINFRA”: http://aginfra.eu, OpenAIRE: https://www.openaire.eu). 6) Storing and preserving metadata over a data infrastructure (e.g. agINFRA e-infrastructure), provided with persistent identifiers compliant with known standards (e.g EUDAT or DataCite recommendations).

### A Global Movement for Open Agricultural Data

After the kick-off meeting of the agINFRA project, it was obvious that the expectations were set very high. The agINFRA project and the work performed quickly got in the spotlight of the EC and global initiatives as the Research Data Alliance (RDA; https://rd-alliance.org). Up to now, the agINFRA project has been invited in a number of EC events (e.g. European Union Delegation to Australia on Research Infrastructure) and has an important role in the RDA leading the Interest Group on Agricultural Data and supporting the Wheat Data Interoperability Working Group. In the meantime, the movement of open data for the agricultural domain was spreading with a highlight event the ‘G-8 International Conference on Open Data for Agriculture’ that took place in Washington, D.C., US, on April 2013, just in the middle of the agINFRA project. Following this event, the Global Open Data for Agriculture and Nutrition (GODAN; http://www.godan.info) initiative was launched in October 2013. GODAN is an initiative that seeks to support global efforts to make data relevant to agriculture and nutrition available, accessible, and usable for unrestricted use worldwide. The initiative focuses on building high-level policy and public and private institutional support for open data, encouraging collaboration and cooperation among existing agriculture and open data activities, without duplication, bringing together all stakeholders to solve long-standing global problems. The agINFRA project was among the first to support GODAN and both the project and the project’s partners are actively involved in the initiative. The agINFRA services and outcomes in many times have set the pace for the GODAN strategy towards agricultural open data. In collaboration with CIARD, agINFRA supported the GODAN initiative and collaborated for the organization of its annual meetings and workshops.

### Conclusion: Creating new products and opportunities

**Figure 3.**
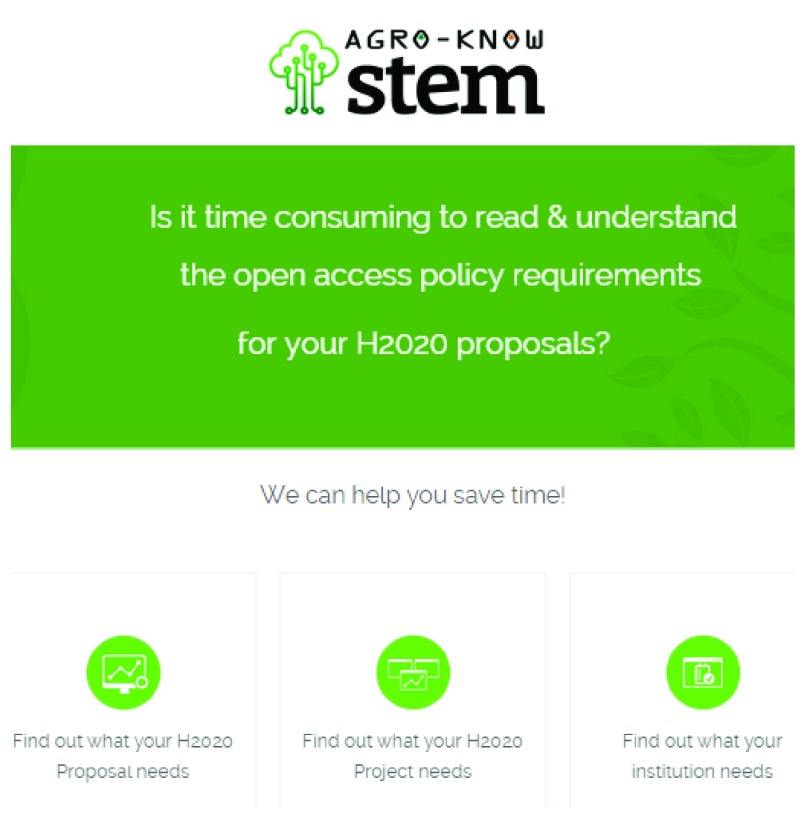
The landing page of the Agro-Know Stem. Source: Agro-Know Stem website ( www.akstem.com).

agINFRA provides data infrastructure and services for agricultural and related scientific communities, relevant data repositories in Europe, as well as virtual collaboration with researchers and repositories from other countries around the globe. This effort was realized with a European approach, involving partners from EU member states and European and international initiatives. agINFRA had identified a strategy to deliver socio-economic impacts across Europe. Towards this end, two different aspects of agINFRA work implemented this strategy: the open availability of services to create new products and the organisation of hackathons events to train users and create new opportunities ^2^. Firstly all services created and data managed by agINFRA are openly shared with the community so anyone can use them. Today business models around open data and open services and are more and more common. In agINFRA one of the partners, Agro-Know, took advantage of the provided technology to create and launch a new product, the Agro-Know Stem ( http://www.akstem.com), a solution that help users open, track, monitor and disseminate their research data. The Agro-Know Stem makes use of many of the agINFRA technologies (e.g. CIARD-RING, micro-finders, Grid powered aggregation workflows, etc.) and experience (e.g. metadata formats, vocabularies) to provide a high end product to their clients ( Figure 3).

Similarly and in order to stimulate the community for the creation of new products/applications, the agINFRA project organized, sponsored or supported a number of hackathons events around the world. A hackathon is an event, which aims to use datasets from various sources (agricultural sciences in the case of agINFRA) in order to provide alternative, useful applications or enhance existing ones with new functionalities. It brings together web/software developers, domain and usability experts who are interested in applying their technical skills in the agricultural context and use the data sets suggested by the organizers in order to develop useful applications. Typical hackathons focus on the provision of working prototypes for one or more problems proposed by the organizers. The outcomes from hackathon events are propriety of the participants who are up to them to use this chance to evolve their idea to actual products. In the context of the agINFRA hackathons, new ideas and applications were conceptualized with some of them aiming to become products (e.g. the Skalidi project, an outcome of the Athens Green City Hackathon).
