# Supplementary material for: agINFRA: a research data hub for agriculture, food and the environment
Source: F1000Res. 2015 Jun 15;4:127. Originally published 2015 May 26. [Version 2] doi: 10.12688/f1000research.6349.2 (PMC4544381; doi:10.12688/f1000research.6349.2)
Supplement: Peer review history

# Peer review: Referee response for version 2

**DOI:** 10.5256/f1000research.7112.r8761

**Published:** 2015-08-18

**Authors:** Christopher Brewster

**Affiliations:** 1 Aston Business School, Aston University, Birmingham, UK

This paper is a good summary of the objectives and achievements of the AgInfra project,

and as such provides an good introduction for researchers wishing to use the AgInfra tools and services.

The paper could be strengthened by:

1. The description of 1-2 tools developed within the project so that the reader can understand what was possible after the project that was not before.
2. The "conclusion" section should be left with the title "Creating new products and opportunities" and a separate conclusion added which could a. reflect on where the data infrastructure for agrifood research is heading now, and/or b. identify directions for future research/development project.

Specific Comments

Frequent infelicities in the use of English:

- In several places the language is a little too telegraphic, appropriate for a bullet point list but not very readable for a journal paper.
- What do you mean by "Provide mediating capabilities ..." p2 ?
- "took upon" --> "undertook" p3
- vo.aginfra.eu does not respond
- voms.ipb.ac.rs does not respond
- p3 "and on user membership" --> "and for ..."
- p3 "Both of the generic versions of the SG have been customized to support the needs of the agricultural research data community." -- in what way?
- p3 "allows the easy of new resources to be committed to the project" --> "allows new resources to be easily committed ..."
- p5 "As it has been show cased" --> "As has been ..."
- p5 "The usage of such format" --> "The use of such a format ..."
- p5 "a lot of effort was made on" --> "a lot of effort was devoted to ..."
- p5 "OpenAIRE (https:// www.openaire.eu), a three-year project, establish" --> "established/establishes (?)"
- p5 "European Resuscitation Council Guidelines on Open Access" -- are you sure it is "resuscitation"? Repositories? Resuscitation does not make sense.
- p5 "dissemination aiming among others to provide" --> "dissemination, with the aim to provide"
- p5 "These recommendations are: ..." --- there are no verbs in the recommendations which follow so it is unclear what the recommendations are. I suggest the use of modal verbs such as "should/ought/might/may ... do XYZ"
- p5 concerning the recommendations - should the recommendations give *examples* or should they be more categorical?
- p5 "After the kick-off meeting of the agINFRA project, it was obvious that the expectations were set very high. " -- irrelevant/inappropriate
- p6 - all the material explaining what GODAN is feels extraneous to the project. A short reference should suffice.
- p6 "This effort was realized with a European approach ..." -- what does this mean?
- p6 "Towards this end, two different aspects of agINFRA work implemented this strategy" --> "In order to implement this strategy, agINRA undertook two types of actions:"

I have read this submission. I believe that I have an appropriate level of expertise to confirm that it is of an acceptable scientific standard.

# Peer review: Referee response for version 1

**DOI:** 10.5256/f1000research.6807.r8763

**Published:** 2015-06-03

**Authors:** George Adamides

**Affiliations:** 1 Agricultural Research Institute, Nicosia, Cyprus

In general I find the article very interesting and its tackling a very interesting topic sharing open scientific data.The article is well organized and provides sufficient information on the goals and outcomes of the agInfra project. I would expect to see more on related work and other background information related to the sharing of agricultural information among scientist. For example what other practices exist and how the agINFRA project outcomes complement or differ from other sharing tools for scientific research (not necessarily only in agriculture).

I have read this submission. I believe that I have an appropriate level of expertise to confirm that it is of an acceptable scientific standard.

# Response

**Published:** 2015-06-09

**Authors:** Andreas Drakos

Dear George,

thank you very much for your comments. The main concept of the agINFRA project was to re-use main infrastructure elements, connect with existing generic solutions and create something specified for the agricultural research domain. While in this article we tried to summarize the  work of agINFRA,the specific aspects of each element has been presented in the numerous publications made from the partners (they can all be found at http://publications.aginfra.eu/ ).

The agINFRA vision was to improve research collaboration and data exchange, but during the lifetime of the project, this had to be limited in creating the necessary building blocks (e-infrastructure) for services to built upon (as for example the new AGRIS). Hopefully this effort will continue and in the future we will be able to present a number of different end-user services specialized for the agricultural research community.

# Response

**Published:** 2015-06-09

**Authors:** Vassilis Protonotarios

**Affiliations:** Agro-Know, Greece

Dear George,

We would like to thank you for your comments. As Andreas mentioned earlier, the agINFRA project aimed at reusing existing components and provide the necessary interconnections or missing links in order to provide meaningful services that would facilitate agricultural researchers in conducting, publishing and disseminating their research.

Despite the fact that we could indeed elaborate on the existing similar efforts, previous experiences and use cases, we decided to focus on the agINFRA approach and how potential end-users could benefit from that. However, based on your idea, we could work on an additional article that will focus on the background of the agINFRA project and the services that the project made use of.
